# Ultrahigh Frequency Ultrasonic Transducers Design with Low Noise Amplifier Integrated Circuit

**DOI:** 10.3390/mi9100515

**Published:** 2018-10-12

**Authors:** Di Li, Chunlong Fei, Qidong Zhang, Yani Li, Yintang Yang, Qifa Zhou

**Affiliations:** 1 School of Microelectronics, Xidian University, Xi’an 710071, China; qdzhang@xidian.edu.cn (Q.Z.); yanili@mail.xidian.edu.cn (Y.L.); ytyang@xidian.edu.cn (Y.Y.); 2 Department of Ophthalmology and Biomedical Engineering, University of Southern California, Los Angeles, CA 90089-1111, USA; qifazhou@usc.edu

**Keywords:** ultrahigh frequency ultrasonic transducer, Si lens, tight focus, finite element simulation, low noise amplifier (LNA), noise figure

## Abstract

This paper describes the design of an ultrahigh frequency ultrasound system combined with tightly focused 500 MHz ultrasonic transducers and high frequency wideband low noise amplifier (LNA) integrated circuit (IC) model design. The ultrasonic transducers are designed using Aluminum nitride (AlN) piezoelectric thin film as the piezoelectric element and using silicon lens for focusing. The fabrication and characterization of silicon lens was presented in detail. Finite element simulation was used for transducer design and evaluation. A custom designed LNA circuit is presented for amplifying the ultrasound echo signal with low noise. A Common-source and Common-gate (CS-CG) combination structure with active feedback is adopted for the LNA design so that high gain and wideband performances can be achieved simultaneously. Noise and distortion cancelation mechanisms are also employed in this work to improve the noise figure (*NF*) and linearity. Designed by using a 0.35 μm complementary metal oxide semiconductor (CMOS) technology, the simulated power gain of the echo signal wideband amplifier is 22.5 dB at 500 MHz with a capacitance load of 1.0 pF. The simulated *NF* at 500 MHz is 3.62 dB.

## 1. Introduction

Ultrahigh frequency ultrasound has recently been investigated as a tool in the field of microbiology. Applications include acoustic microscopy for the non-invasive investigation of biological tissue and living cells [1,2,3,4] and non-contact manipulation of microparticles or cells that are based on radiation force principle [5,6,7]. State of the art in acoustic microscopy is to work with single element focusing transducers. In most cases, the transducers in the ultrahigh frequency range are based on ZnO thin films on sapphire with a grind spherical cavity as a focusing element on the opposite side of the ZnO layer. 

The attenuation of generated signal in water is proportional to the covered distance and the square of the frequency. With the increasing operation frequency, the focus distance of the transducer should decrease, thus demand smaller radius and higher sphericity of the lens. When comparing with a sapphire lens for ultrahigh frequency ultrasonic transducer design, a silicon lens might be more appropriate for the following reasons: (1) the silicon wafer is cheaper than the sapphire crystal; (2) good uniformity can be utilized using microelectromechanical systems (MEMS) lithography and etching techniques for the silicon lens rather than the grinding method for the sapphire lens; and, (3) it is possible to make multi lens on a silicon lens body for advanced transducer configurations. In addition, the signal amplitude of the ZnO based transducer is rather low for a good performance in acoustic microscopy due to the weak piezoelectric behavior. Another important non-ferroelectric piezoelectric material, Aluminum nitride (AlN), possess better chemical and thermal stabilization, better compatibility with the complementary metal oxide semiconductor (CMOS) technology than ZnO [8,9,10]. Furthermore, the much higher longitudinal wave velocity benefits AlN for ultrahigh frequency application.

**Figure 1:**
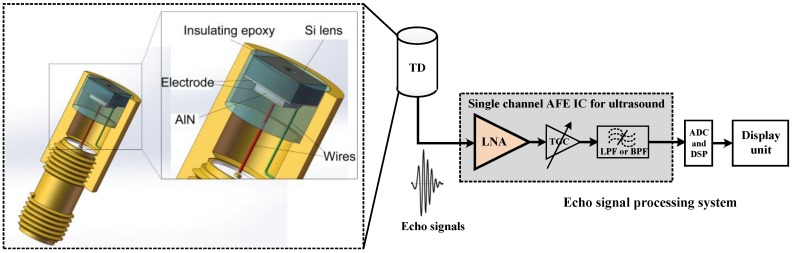
Schematic diagram of the AlN ultrahigh frequency ultrasonic transducer and the echo signal processing system.

Figure 1 shows a schematic diagram of an ultrahigh frequency ultrasonic transducer that is based on the silicon acoustic lens. The ultrasound is generated by an AlN thin piezoelectric layer with electrode on both sides. The AlN layer is sputtered on the silicon lens body according to special designed pattern to reduce the rim echo around the lens cavity. Two lead wires are electrical connected with the bottom and top electrodes. The whole device was encased in a brass tube to provide RF shielding. The gap between the brass tube and the device was filled by insulating epoxy. 

Miniaturization and performance improvement of the ultrasound system have been developed in the past several years. One of the driving forces is the improvement of the transducer technology, and the other one is the advanced semiconductor technology based on which the integrated circuits (IC) for ultrasound application could further enhance the system sensitivity and reduce the cost. The transducer front-end, especially the analog receiving portion, plays a significant role in the overall performance of the system. Low noise, large bandwidth, high frequency, and linearity are the important aspects that should be considered carefully. The typical ultrasound receiving analog front-end (AFE) IC, as shown in Figure 1 consists of a low noise amplifier (LNA), a time-gain-compensation (TGC) amplifier and a low-pass or band-pass filter, and generally these blocks are arranged in a cascade scheme to make up the AFE receiver chain [11]. The amplified and filtered echo signals will be finally converted to be digital signals by an analog-to-digital converter (ADC) and processed by the digital signal processing (DSP) block. In fact, performances of the first block LNA including bandwidth, noise figure, gain and linearity have a decisive impact on the performances of the overall AFE receiver chain. The noise figure (*NF*) of an *n*-cascaded structure receive chain can be expressed as

(1) $$NF=NF_{1}+\frac{NF_{2}-1}{G_{1}}+\frac{NF_{3}-1}{G_{2}}+\cdots +\frac{NF_{n}-1}{G_{n-1}}$$

where *NF_i_* and *G_i_* stands, respectively, for the noise figure and gain of the *i*th circuit block in the chain. It is obvious that high gain (*G*_1_) of the LNA could reduce the noise contribution of the following stages, and low noise figure (*NF*_1_) of the LNA could result in a low *NF* of the whole receiver chain. Meanwhile, high gain of the LNA could also relax the circuit complexity of the other gain blocks in the AFE. Since the real medical ultrasound echo signal is not a signal with only one single frequency, large bandwidth of the first block LNA is desirable to guarantee the integrity of the information carried in the ultrasonic echoes. The linearity of the LNA should also be considered and simulated carefully since the distortions and nonlinearities introduced by the LNA are unlikely to be removed by the following stages in the AFE [12,13,14]. Although some well-known chip design companies, such as the ADI (Analog Devices Inc., Norwood, MA. USA), MAXIM (MAXIM integrated Inc., Sunnyvale, CA, USA), and TI (Texas Instruments Inc., Dallas, TX, USA) had designed a series of low noise LNA chips which can be used for the ultrahigh frequency ultrasonic applications, a good trade-off, especially between the noise and gain, were not achieved. Most of the chips were featured with good noise performance while poor gain performance.

In this work, we presented the design of 500 MHz ultrasonic transducers using AlN piezoelectric thin film as the piezoelectric element and using silicon lens for focusing. The fabrication and characterization of silicon lens was presented in detail. As the most important circuit block in the AFE for echo signal processing, a wideband and high gain LNA with an inductor-less CS-CG combination structure was also designed in this work. The LNA that was proposed in this work featured low noise figure, high gain, and good linearity characteristics.

## 2. Fabrication and Characterization of Silicon Lens

The isotropic XeF_2_ dry etch process was chosen for etching silicon cavity over the conventional isotropic wet etch. Previously we used HF:HNO_3_:CH_3_COOH = 1:2:3 (HNA) solution wet etching [3]. The dry etch process has several advantages over it: (1) photoresist can be directly used as etching mask while an additional hard mask (SiN) that is grown by low pressure chemical vapor deposition LPCVD is necessary in the wet etch process. Therefore, fabrication time is reduced. (2) XeF_2_ etching can be realized at a slower etching rate than wet etching, leading to better half sphere shape and better surface smoothness. (3) XeF_2_ etching process is more controllable. We can control the etching depth easily by just changing the number of etching cycles. Contrarily, wet etching is very sensitive to the HNA solution’s composition (the ratio of hydrofluoric acid, nitric acid, and acetic acid), which cannot be controlled accurately. (4) All reactions happen inside closed chamber which enables people to avoid handling toxic or corrosive chemical.

The reactant, XeF_2_, is in a solid crystalline form at room temperature. When exposed to low pressure, the XeF_2_ crystal sublimates to gas phase. It has high selectivity on silicon over other materials, such as most photoresist, oxides, nitrides, and many metals. The chemical reaction involved is:

2XeF_2_ + Si→ 2Xe (g) + SiF_4_ (g) (2)

Photolithography was used to transfer patterns onto photoresist for the fabrication of the cavity. The mask pattern designed is 4 mm × 4 mm arrays of circles with diameter ranging from 50 μm to 300 μm (25 μm step from row to row) in order to obtain silicon lens of different size. The photolithography process is: Firstly, the silicon wafer was spin coated photoresist (AZ MIR701, 3000 rpm, 40 s, postbake: 90 °C, 1 min; MicroChemicals GmbH, Ulm, Germany). Then mask aligner was used to expose the coated wafers for 20 s at power of 3.75 mW/cm^2^ (postbake 110 °C, 1 min). Next, the exposed wafer was developed by an AZ 300 developer for one minute, and the pattern was successfully transferred onto the photoresist. The wafer was put into XeF_2_ etcher chamber and went through 125 etch cycles (about 4 h). At last, residual coating on samples was removed by acetone with ultrasound agitation.

**Figure 2:**
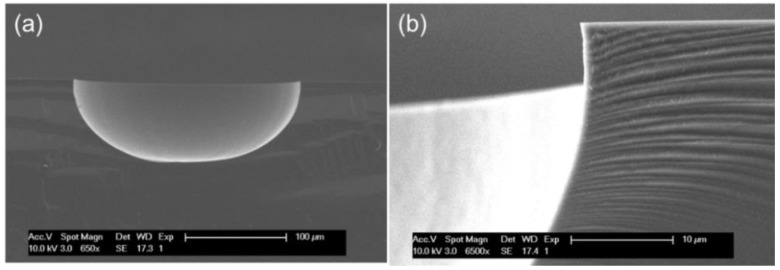
(**a**) Scanning electron microscope (SEM) image of a cross section of a dry etched Si cavity; (**b**) SEM image of a corner of a dry etched Si cavity.

The final diameter of silicon lens ranges from 200 μm to 540 μm, depending on the original mask pattern size. Figure 2 shows a cross section of the silicon lenses and a zoom-in image of a corner of the lens, by which we can inspect the shape and surface smoothness. As can be seen, the hemispherical shape is clear and the surface smoothness is at hundred nanometer level.

## 3. Transducer Design and Finite Element Simulation

Aluminum nitride was selected for piezoelectric layer of the ultrahigh frequency transducer duo to it excellent properties, such as a high longitudinal velocity (~11,000 m/s), high thermal stability (melting point ~2100 °C and piezoelectric effect application up to 1150 °C), relatively high electromechanical coupling coefficient ((*k_t_* ~ 0.28), and low dielectric constant (*ε^s^*/*ε* ~ 8). Furthermore, AlN is compatible with the complementary metal oxide semiconductor (CMOS) technology. Specific design parameters and performance of the transducer were simulated through a finite element model-based simulation software PZFlex (PZFlex2016, Weidlinger Associates, Inc., Mountain View, CA, USA). The main materials that were used for the simulation are listed at Table 1.

**Figure 3:**
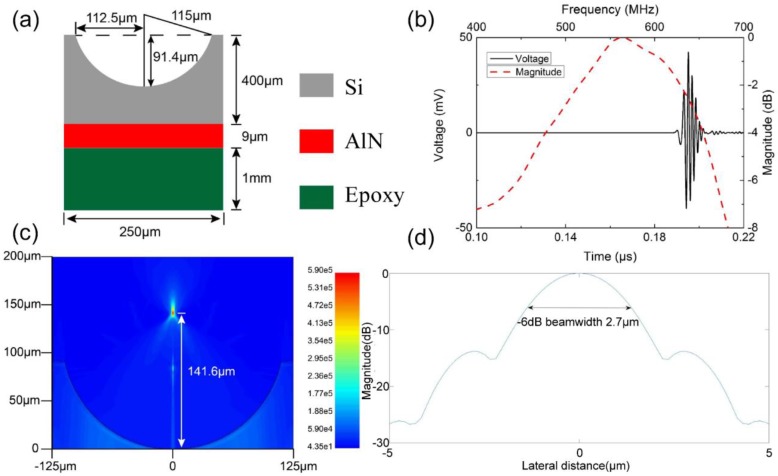
(**a**) Design specifications of the Aluminum Nitride (AlN) stack with lens and backing material; (**b**) the simulated pulse-echo waveform and frequency spectrum of the silicon lens transducer; (**c**) the acoustic pressure pattern generated by the transducer; and, (**d**) The on focus lateral beam profile of the silicon lens transducer.

Figure 3a gives the designed specification of the AlN stack together with the lens and backing material. The thickness of AlN film was 9 μm in order to achieve center frequency of 500 MHz. AlN film was connected series to a 50 Ω resistor during the simulation process, and the transducer was driven by a sinusoidal signal with excitation frequency of 500 MHz and peak-to-peak voltage of 1 V. Box size was chosen to be 1/20 wavelength at both the axial and lateral direction. Simulation time was set to be 0.22 μs for signal sending and receiving. Figure 3b shows the pulse-echo waveform and frequency spectrum that were achieved from the finite element simulation. The center frequency (*f_c_*) and −6 dB bandwidth (*BW*) were determined by the following equations:

(3) $$f_{c}=\frac{f_{l}+f_{u}}{2}\;$$

(4) $$BW=\left(\frac{f_{u}-f_{l}}{f_{c}}\right)\times 100\%$$

where *f_l_* and *f_u_* are defined as lower and upper −6 dB frequencies, respectively, at which the magnitude of the amplitude in the spectrum is 50% (−6 dB) of the maximum. The center frequency and −6 dB bandwidth were calculated to be 559 MHz and 40%. In the simulation, the focal depth was determined from the acoustic pressure pattern (Figure 3c). The focal depth of the AlN transducer was calculated as 143.6 μm, assuming a value of 1490 m/s for the speed of sound in water. The on focus lateral beam profile (Figure 3d) demonstrated the −6 dB beam width simulated to be 2.7 μm. The finite element simulation results demonstrate that, based on this AlN transducer with silicon lens, it is possible to design and fabricate ultrasonic transducer with high center frequency and narrow −6 dB beam width. 

## 4. The Echo Signal LNA Integrated Circuit Design

**Figure 4:**
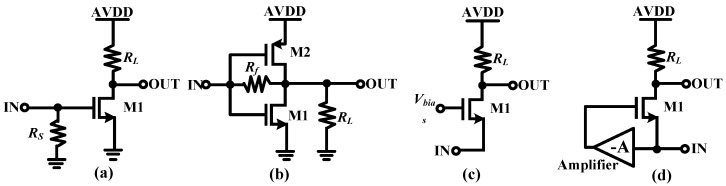
Inductor-less wideband low noise amplifier (LNA) (**a**) common-source (CS) amplifier (**b**) shunt-feedback (SFB) amplifier (**c**) common-gate (CG) amplifier and (**d**) CG amplifier with gm-boosting technique.

The LNA used for the ultrasound echo signals processing should be featured with low noise figure, wideband, high gain, and good linearity characteristics, as mentioned in the first section. These performance requirements can be well met by the traditional inductor-based LNAs. However, the on-chip bulky inductors occupy very large area which counters the purpose of high integration required in the ultrasound systems and many other applications. In addition, accurate inductor models are very difficult to build, which may lead to many times of tape-out and thus greatly increasing of the cost [15,16,17,18]. Therefore, inductor-less LNA has become more attractive in these years and several topologies had been proposed in the published literatures [19,20,21,22,23,24,25]. These topologies can be in fact divided into three categories, including common-source (CS) structure with resistor-terminated [15,26], shunt-feedback (SFB) amplifier [27], and common-gate (CG) structure with capacitive cross-coupling or gain boosting techniques [28,29,30,31]. The resistor-terminated CS scheme as shown in Figure 4a provides the input impedance by using a 50-Ω shunt resistor. However, large transconductance (*g_m_*) of input transistor (M1) is needed to achieve low noise performance. For both the SFB (Figure 4b) and CG (Figure 4c) schemes, low noise figure can be achieved with small *g_m_* of the input transistor, but the power consumption is generally high to achieve the input matching. The *g_m_*-boosting technique is popular in these years and its basic idea is using an auxiliary voltage gain to simultaneously apply signal on both gate and source of the input transistors (Figure 4d). The *g_m_* of the CG transistor (M1) can be boosted, since it forms a negative feedback loop with the amplifier. This technique offers a low noise figure of the LNA and meanwhile a favorable power consumption-input matching tradeoff.

**Figure 5:**
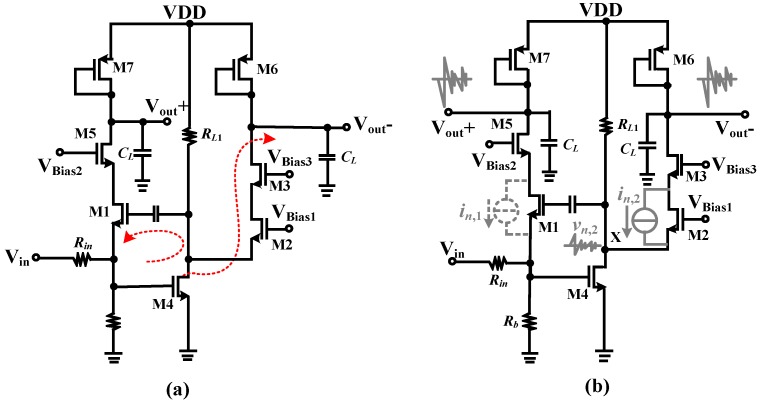
The proposed LNA (**a**) single-end schematic and signal propagation paths and (**b**) noise model of the key devices and analysis of noise cancelation mechanism.

Inductor-less scheme and CS-CG combination structure are employed in this work for the LNA integrated circuit design to meet the small chip area and high performance requirements. The generations of CMOS technologies exhibit excellent performances, such as low noise figure, high characteristic frequency, and so on, and could also provide larger margin for the design of high performance integrated circuits with low cost. The medical input ultrasound signal frequency in this work is centered at 500 MHz, and since most current CMOS processes can handle this easily, a 0.35 μm CMOS process is adopted for the LNA design with better integration and power reduction being achieved. The single-end schematic of the proposed LNA in this work is shown in Figure 5a. The input resistor *R_in_* is the source impedance and it equals typically 50 Ω. The first stage is in fact a CG amplifier using *g_m_*-boosting technique. The active feedback amplifier is realized by a common-source amplifier consisted by transistor M4 and load resistor *R_L_*_1_, and the gain of the amplifier can be expressed as −*g_m_*_4_*R_L_*_1_. If *A_v_* is expressed as the local open loop gain, the impedance matching can be achieved when

(5) $$R_{in}=\frac{1}{g_{m1}\left(1+A_{v}\right)}$$

where *g_m_*_1_ is the transconductance of the feedback transistor M1. Therefore, when compared with the traditional common-mode or common-gate structure, the transconductance of the LNA needed for the input impedance matching can be reduced by a factor of (1 + *A_v_*) [32]. Since a fully differential scheme will be adopted in this design, the circuit could also provide a negative gain for the negative output *V_out_*_−_ to form a positive feedback when the whole differential circuits are realized. The input signal is firstly amplified by the CS transistor M4 and then injected into the CG amplifier consisted by M2, M3, and diode transistor load M6. The folded-cascode structure is also employed in this design where transistor M5 is stacked on the top of M1 and M3 on the top of M2 to provide high reverse isolation and therefore the power gain. For the positive gain path from *V_in_* to *V_out_*_+_, the gain can be expressed as

(6) $$A_{v,out+}=g_{m1}\left(1+g_{m4}R_{L1}\right)\left(g_{m5}r_{O5}r_{O1}||\frac{1}{g_{m7}}\right)\;$$

and for the negative gain path from *V_in_* to *V_out_*_−_

(7) $$A_{v,out-}=-g_{m4}R_{L1}g_{m2}\left(g_{m3}r_{O3}r_{O2}||\frac{1}{g_{m6}}\right)$$

where *r_Oi_* are drain-to-source resistance of transistor M*i*.

In the proposed LNA, noise contributions from transistors M1 and M2, as shown in Figure 5b can be canceled. Taking noise contribution from M2 as the example and similar analysis can also be applied for the one from M1. The noise that is generated by transistors M2 can be modeled as a current source *i_n_*_,2_, which will both generate a noise voltage *v_n_*_,2_ at point X and the negative output *v_n,out_*_−_, which can be given by

(8) $$v_{n,out-}=-\frac{i_{n,2}}{g_{m6}}$$

The *v_n_*_,2_ will be also amplified by the CS amplifier consisted by M1, M5, and M7, and the noise voltage at the *V_out_*_+_ end can be given by

(9) $$v_{n,out+}=-i_{n,2}R_{L1}g_{m1}\left(g_{m5}r_{O5}r_{O1}||\frac{1}{g_{m7}}\right)$$

The noise contribution from M2 can be cancelled when *v_n,out_*_+_ = *v_n,out_*_−_, since it becomes a purely common-mode signal and it will finally undergo subtraction at the output ends *V_out_*_+_ and *V_out_*_−_. Therefore, parameters of the related devices in the circuits should be designed to satisfy

(10) $$R_{L1}g_{m1}g_{m6}\left(g_{m5}r_{O5}r_{O1}||\frac{1}{g_{m7}}\right)=1$$

Noise cancelation mechanism greatly improves the noise performance of the whole circuits [32,33]. The thermal noise of resistor *R_L_*_1_ and channel thermal noise of transistor M4 then take up the primary part of the whole LNA noise. 

High order harmonic distortions have a much smaller contribution to the nonlinearities of the LNA due to their low power while the low order ones, especially the 2nd and 3rd harmonics are the prominent components that should be considered. Fully differential structure has the advantage of ideally canceling the even order harmonics, which can be considered as common-mode components that appeared at the balanced differential output ends. The 3rd harmonics distortion components can be partly cancelled in this work. The distortion currents of the transistors can be modeled as current sources paralleled with the transistors and the distortions from M1 and M2 can be eliminated by the similar mechanism of noise canceling. Attention should be paid that the distortion and noise cancelation might lose effect at a very high frequency due to the phase shift.

**Figure 6:**
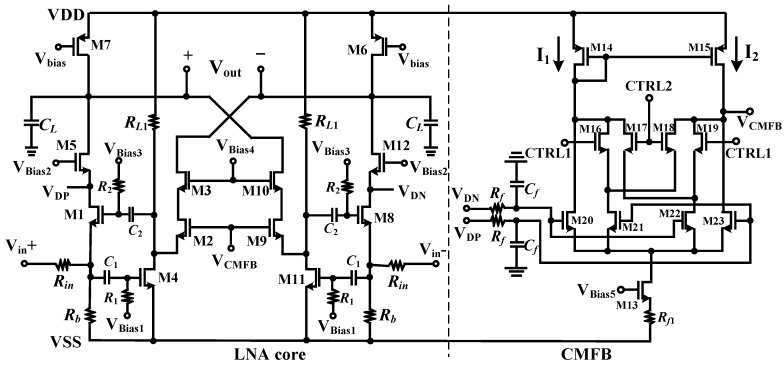
Schematic of the whole LNA with common-mode feedback.

The schematic of the whole differential LNA that was used in this work is shown in Figure 6. Fully differential and symmetrical scheme will not only double the gain, but also achieve a better common mode noise rejection and overcome the performance deterioration of the analog front-end circuits resulted by noise coupling through the substrate from the digital circuits. Bias voltages V_Bias1_~V_Bias5_ are provided through bias resistors *R*_1_ and *R*_2_ to make sure that the transistors in the circuits could operate in saturate states. The coupling capacitances *C*_1_ and *C*_2_ are designed to be much greater than the parasitical capacitances of the input transistors. The cross-coupled scheme at the output ends could further enhance the voltage gain of the differential LNA, and the gain of the differential circuits can be expressed as

(11) $$A_{v,diff}=g_{m1}\left(1+g_{m4}R_{L1}\right)\left[\frac{1}{g_{m7}}||r_{O5}||\left(g_{m10}r_{O10}r_{O9}\right)\right]+g_{m4}R_{L1}+g_{m2}\left[\frac{1}{g_{m6}}||r_{O12}||\left(g_{m3}r_{O3}r_{O2}\right)\right]$$

Common-mode feedback (CMFB) circuits consisted by transistors M13–M23 is also employed to stabilise the dc operating voltages of the LNA core. It provides a common mode feedback voltage for the gates of M2 and M9 after detecting the common mode voltages of *V_DP_* and *V_DN_*. The voltage test points are located at the drains of M5 and M8 rather than the output ends (*V_out_*_+_ and *V_out_*_−_) for not introducing noise in the outputs. Simple low-pass filters consisted by *R_f_* and *C_f_* are used to remove the high frequency noise. CTRL1 and CTRL2 are two control signals with complementary phases, and transistors M16 to M19 are acted as switches under these two control signals. When CTRL1 is high and CTRL2 is low, the two inputs of the CMFB block are in fact both the sum of the DC voltages of *V_DP_* and *V_DN_* (the AC components are filtered by the RC filters). If the common-mode voltage, for example, is higher than the expected one, the *V_CMFB_* and the currents flowing through M2 and M9 will be decreased. Then, the currents flowing through M5 and M12 will be increased and the common-mode voltage of *V_DP_* and *V_DN_* decreased. Since a current mirror is constructed by transistors M14 and M15, Currents I_1_ and I_2_ on the two branches in the CMFB are equal and the difference between input DC voltages that are applied onto the gates of M20–M23 can then be detected. Therefore, when CTRL1 is low and CTRL2 is high, the CMFB will detect the difference between the DC voltages of *V_DP_* and *V_DN_*. With the feedback loop, the DC offset will then be corrected. 

**Figure 7:**
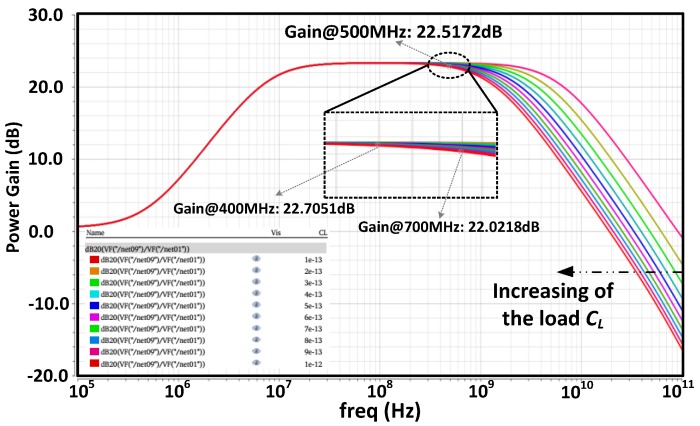
The simulated AC response of the LNA with different *C_L_*.

The proposed differential LNA was designed by using a 0.35 μm CMOS technology and it consumes a current of 2.5 mA from a 3.3 V power supply. Generally, the capacitance load of the LNA (*C_L_* in Figure 6) which is also the input capacitance of the next stage in the AFE chain may vary over a certain range, and therefore simulations of the gain and bandwidth with different capacitances load should be considered. Figure 7 shows the simulated AC response of the LNA with the capacitances load tuning from 0.1 pF to 1.0 pF. The bandwidth decreases with the increase of the *C_L_*. The maximum gain of the LNA is about 23.2 dB, and at 500.023 MHz the gain is about 22.5 dB. The gain variation retains a flatness of smaller than 0.7 dB over the frequency range from 400 MHz to 700 MHz with a capacitance load of 1.0 pF.

**Figure 8:**
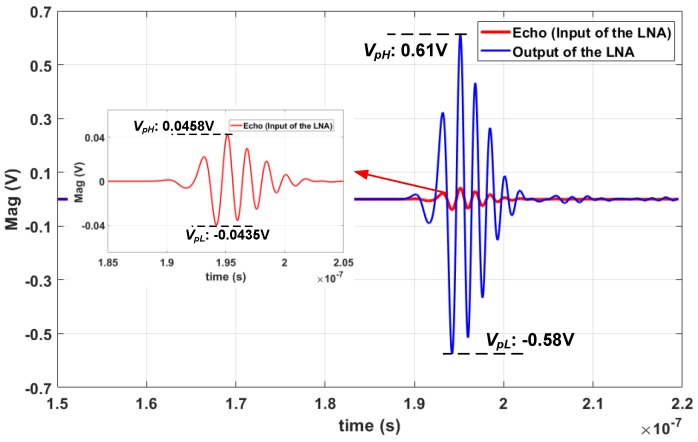
Transient simulation waveform of the LNA output.

**Figure 9:**
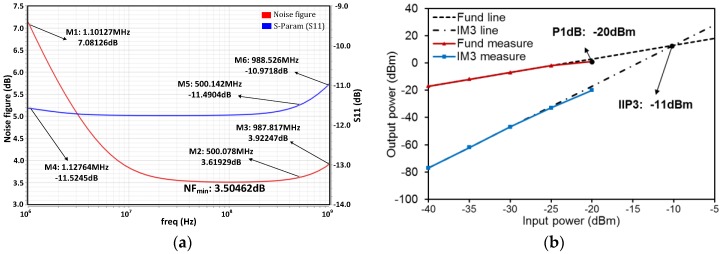
Simulated results of (**a**) *NF* and S11 versus input frequency (**b**) distortion performance in terms of the input-referred P_1dB_ and IIP3.

Figure 8 presents the transient simulation result of the LNA. The peak-to-peak amplitude of the input signal is about 90 mV and the fundamental frequency 500 MHz. After being amplified by the LNA, the magnitude is from −0.58 to 0.61 V and the power gain is about 22.43 dB, which is consistent with the AC simulation results that are shown in Figure 7. Figure 9a presents the simulated noise figure (*NF*) and input reflection coefficient (S11) of the LNA versus the input frequency. The *NF* is about 3.62 dB at 500 MHz and the minimum is about 3.5 dB from 0.1 to 1 GHz. The increase of *NF* at low frequencies is due to the flicker noise (1/*f* noise), and due to the drop in gain, it increases at high frequencies. The S11 is lower than −10 dB over the bandwidth, which implies that good matching performance of the LNA input is achieved. Two-tone test is done for measuring the input 1 dB compression point (P_1dB_) and third-order intermodulation (IM3) distortion of the LNA, which are shown in Figure 9b. The P_1dB_ and the input third-order intercept point (IIP3) at 500 MHz are respectively −20 dBm and −11 dBm, which imply that the LNA could accommodate input echo signals with large amplitudes and linearity performance. 

## 5. Conclusions

In this work, ultrahigh frequency ultrasonic transducers are designed using AlN piezoelectric thin film as the piezoelectric element and using silicon lens for focusing. The fabrication and characterization of silicon lens was presented in detail. Finite element simulation was used for transducer design and evaluation. The results demonstrate that, based on this AlN transducer with silicon lens, it is possible to design and fabricate ultrasonic transducer with high center frequency and narrow −6 dB beam width. A wideband inductor-less LNA with CS-CG combination structure for the ultrasonic medical echo signal processing was also proposed in this work. Active feedback structure and noise cancelation mechanism were employed and the LNA featured wideband coverage while maintaining low noise figure, high gain, and good linearity. Designed by using a 0.35 μm CMOS technology, the simulation results show that the LNA achieves a power gain of 22.5 dB at 500 MHz and remains a gain flatness of smaller than 0.7 dB over a frequency range from 400 MHz to 700 MHz. The simulated noise figure is 3.62 dB at 500 MHz, and the P_1dB_, IIP3 at 500 MHz are, respectively, −20 dBm, −11 dBm.

## Figures and Tables

**Table 1:** Materials used for the transducer simulation consideration.

Material	Function	c (m/s)	ρ(kg/m^3^)	Z(MRayl)
AlN	Piezoelectric element	11,000	3260	35.86
Si	Lens	8430	2340	19.8
Water	Front load	1540	1000	1.54
EPO-TEK 301	Backing	2650	1150	3.05
